# Report upon the Mortality of Lunatics

**Published:** 1841-07-01

**Authors:** 


### REPORT UPON THE MORTALITY OF LUNATICS. By *William Farr*, Esq. F.S.S. Read before the Statistical Society of London.

MR. FARR is well known to all our readers as a very zealous and able cultivator of medical statistics. The Report before us is as creditable to his diligence and judgment as its predecessors, and has been drawn up, we perceive, at the request of the Council of the Statistical Society. It is founded on the Reports of the Hanwell Asylum, Returns from the Bethlem Hospital, and the valuable series of tables submitted to the Society last year by Colonel Sykes. It was thought desirable that the mortality of lunatics in two of the largest public institutions of the country, should be compared with the mortality in the licensed proprietary houses; and that, if the mortality differed, the differences should be investigated, and traced to their causes, by the methods of statistical analysis which we now possess.

We shall present a pretty full account of this Report, as we think it likely to prove serviceable, more particularly as Mr. Ewart has given notice of his intention to move in the House of Commons for the appointment of a fresh Committee to inquire into the management of the Asylums.

The persons of unsound mind in England amount to several thousands. They are usually of middle age, frequently parents, and are of all conditions and ranks of life: 494 lunatics confined under the Crown possess property yielding an annual income of 317,154*l.** 6,402 idiots, and 7,265 lunatics, have been returned to Parliament as paupers.

" Great improvements have taken place in the treatment of lunatics. In the best asylums they are no longer shut up in cells like wild beasts, nor punished

---

* *See* Parliamentary Return, Session 1839, No. 378.

by harsh keepers. Their chains have gradually been struck off. A further step has been attempted. At the Middlesex Asylum no strait-waistcoats, straps, or other instruments of personal coercion have been used since the 21st of September, 1839. The experiment was first tried at Lincoln, and it is now contended by persons of experience, ability, and integrity—by Mr. Hill, Dr. Conolly, and the visiting justices of Middlesex,—that in a house properly built, with skilful medical supervision, and a sufficient number of humane and intelligent keepers, personal coercion should be abolished. This is denied by other gentlemen of equal humanity, who maintain that although all restraint *may* be dispensed with, the strait-waistcoat should still be employed as a remedy in the paroxysms of mania. A keen controversy has been waged on the subject. Asylums not only differ widely in the extent to which restraint is carried, but in the space allotted to patients, in their employment, food, and medical treatment. The cost of criminal lunatics at Bethlem is 15*s.* a-week; of idiots or lunatics in the workhouses, 2*s.* 10*d.* to 3*s.* 6*d.* a-week. Some of the asylums are under the control of the visiting justices, others are visited by the Metropolitan Commissioners; the hospitals of Bethlem and St. Luke are not visited at all, but are managed by the officers and governors; while a very large number of lunatics are farmed out, or confined in workhouses, by the parish authorities." 2.

Amongst so many systems the determination of the best can only be effected by a comparison of their results—in fact, of the recoveries and deaths.

The number of lunatics and dangerous idiots under confinement in Middlesex, and in the parts of Surrey and Kent within the jurisdiction of the Metropolitan Commission, is about 3,110, and the following was their distribution in 1839:—

| | Males. | Females. | Total. |
|---|---|---|---|
| In the Asylum at Hanwell . | 346 | 488 | 834 |
| Bethlem Hospital . . . | 148 | 151 | 299* |
| St. Luke's . . . . . | 104 | 136 | 240 |
| Guy's . . . . . . . . | ... | 24 | 24 |
| 34 Licensed Houses . . | 787 | 926 | 1,713 |
| Total . . . | 1,385 | 1,725 | 3,110 |

459 men, and 419 women in the licensed houses are not paupers; and many persons insane in different degrees remain at home under the care of their friends. The London workhouses contain a considerable number of idiots and lunatics. Exclusive of these 3,110 persons, others are confined as lunatics in the public institutions of the metropolis. When it is considered that insanity is a long disease, which not only disables the patient, but often renders him difficult to control, and dangerous to himself and to society, the fact that 7 in 10 of the 3,100 lunatics fall upon the public for support and treatment, will not be deemed surprising.

The Hanwell Asylum was opened on May 16th, 1831, and the number

* Exclusive of 16 out on leave.

of lunatics admitted in the $9\frac{1}{3}$ years, ending September 30th, 1840, as shewn in the following Table, was 2,029; the number discharged was 1,171; of whom 449 had recovered, 66 had been relieved, and 656 had died: 858 remained in the asylum. More than *half* the patients die in Hanwell, and more than *one-third* are cured.

| | | Men. | Women. | Total. |
|---|---|---|---|---|
| Admitted from 16th March, 1831, to 30th September, 1840 .......... | | 1013 | 1016 | 2029 |
| Discharged during the same period, *viz*.... | Cured ...... | 223 | 226 | 449 |
| | Relieved .... | 42 | 24 | 66 |
| | Died ...... | 374 | 282 | 656 |
| Total.............. | | 639 | 532 | 1171 |
| Remaining on 30th September, 1840 .. | | 374 | 484 | 858 |
| Proportion in 100 of patients discharged, *viz.*— | | | | |
| | Cured.......... | 37. | 42. | 38. |
| | Relieved........ | 7. | 5. | 6. |
| | Died .......... | 58. | 53. | 56. |

It has been a question whether the deaths should be divided, as in this case, by the 2,029 patients admitted, or by the 1,171 discharged, in order to obtain the mortality of the cases. It is evident that the latter is the true divisor; for, if the mortality remained the same, the probability is that the 858 patients *to be discharged* would, *cæteris paribus*, be discharged cured, relieved, and dead, in the same proportions as the 1,171 already discharged.

The average number of lunatics in the Hanwell Asylum, since it was opened, has been about 589, or 250 males and 339 females.

The deaths in the $9\frac{1}{3}$ years ending 30th September 1840 were 656 (males 374, females 282); and the insane population out of which they occurred was = 5,498 living one year; the males 2,334, and females 3,164. The average number of males resident was = 250, and 250 × 9·34 years, the term of residence, = 2,334 years of life. The *annual mortality* of the men was 16 per cent., of the women 9 per cent., and of the whole population, without distinction of sex, 12 per cent.

The mean term of residence in the Middlesex Asylum is 4·48 years—an approximation to the average term of treatment.

From Tables which he submits Mr. Farr observes, that nearly equal numbers of men and women are admitted at the County Asylum, (males 1,013, females 1,016); but that the number of women resident is 36 per cent. greater than the number of men (females 339, males 250); because women remain there about 6 years on an average, and men nearly 3·7 years. The men are discharged more rapidly than the women, both by death and recovery. 11 men per cent. were annually discharged cured, or relieved; and only 8 women. This distinction will explain many anomalous facts; and it should always be taken into account in estimating the prevalence of diseases. Thus there may be ten times as many lunatics in civilized, as in barbarous countries and times; not because the tendency to insanity is greater, but because the lunatics live ten times as many months, or years. The tendency to insanity in a class is expressed by the proportion that become insane.

Mr. Farr next compares the licensed houses with the Hanwell Asylum. It seems that out of 100 cases in the licensed houses, there were 30 deaths, while out of the same number of cases in the Hanwell Asylum there were 56 deaths. But it was the reverse with the annual mortality per cent. The annual mortality per cent. at Hanwell was to that in the licensed houses as 100 to 130. For various reasons the patients remain longer in the Hanwell Asylum than in the licensed houses, from which 37 per cent. were annually discharged alive; while 9·4 per cent. were discharged annually, cured and relieved, from the County Asylum. The number admitted during the six years, June 1833-39, into the licensed houses was 5,386; making 278 more than 5,108, the number discharged by death, recovery, or otherwise. There were 1,435 in the licensed houses on 30th June 1833, and 1,713 on 31st May 1839. The number of inmates had increased 19 per cent., and, notwithstanding the erection of Hanwell, the increase bore principally upon paupers, for 202 of the 278 were paupers. Mr. Farr compares these paupers with the paupers in Hanwell.

The comparative mortality was as follows :—

| | Annual Mortality per cent. | Deaths out of 100 cases discharged. | Mean term of treatment, in years. |
|---|---|---|---|
| Paupers in Licensed Houses.... | 21 | 35 | 1·67 |
| " Hanwell .......... | 12 | 56 | 4·48 |
| Other patients in the Licensed Houses ................ | 11 | 23 | 2·15 |

The annual mortality of paupers in the licensed houses is thus shewn to have been excessive ; in fact, the annual mortality of both male and female paupers in the licensed houses was *nearly twice as great as the mortality of paupers at Hanwell, and twice as great as the mortality of other lunatics in the licensed houses.*

It appears that of the licensed houses four are large, the remainder small. Each of the four large houses contained 265 patients on an average, and the annual mortality was 19 per cent. ; in the small houses, containing 17 lunatics on an average, the mortality was 9 per cent., and the annual mortality in the four houses increased with the number of lunatics. It was 16 per cent. in the house No. 18 ; 18 per cent. in Nos. 32 and 33 ; and 23 per cent. in No. 35. Of the higher class of patients, 26 in 100 cases perished in the large houses, and 21 in 100 in the smaller houses, where the term of treatment was somewhat longer.

"*What is the mortality among lunatics in favourable circumstances? Is insanity a fatal disease?*—Upon the latter question there has been a considerable diversity of opinion. Some lunatics live to an advanced age. Of 213 admitted by Dr. Conolly at Hanwell, 15 were aged 60 and upwards, 1 was between 75 and

80; and 58 in 753 at Hanwell had been labouring under the disorder between 20 and 50 years. In 1835 an action (Fisher v. Beaumont) was brought at the York Assizes to recover from the *Providence Assurance Company*, £2,000. insured upon the life of the Rev. Mr. F——. In charging the jury, the judge said that they had to consider whether insanity had a tendency to shorten life? If insanity had such a tendency, they must find for the defendant; if not, for the plaintiff. The medical evidence was conflicting; and the jury, after a short deliberation, found for the plaintiff, on the ground that insanity had no tendency to shorten life!*

"We have no means of ascertaining the mortality of lunatics at large; but the mortality of lunatics in asylums is much higher than the mortality of the general population, and the excess cannot be ascribed entirely, although it may partially, to the confinement, the unwholesomeness, or the usages of mad-houses. The mean age of lunatics in asylums is about 35-40. The average age of the patients admitted at Bethlem, (1830-34) was 36 years (36·2); and the mean age of 213 admitted at Hanwell by Dr. Conolly was 36½. The mortality at the age 30-40 is 1·2, and at 40-50 is 1·5 per cent. in England and Wales. In cities the mortality at a corresponding age is not more than 2 per cent. annually. Now the annual mortality at Bethlem, where dangerous cases are carefully excluded, was 9 per cent., in 1827-39. At Gloucester, one of the county asylums, at which the treatment is most successful, the diet is generous and nutritious, and the patients live as much as possible in the open air,—the annual mortality is 7 per cent.

The annual mortality of severe cases of insanity cannot, I think, in favourable circumstances, be less than 6 per cent.; so that the mortality is three times greater among lunatics, than among the general population, at the same age. We have seen, however, that the annual mortality among the better class of patients in the licensed houses was 11 per cent., among paupers at Hanwell 12 per cent., among paupers in the licensed houses 21 per cent., and among pauper men at one licensed house 27 per cent.;—as high as the rate of mortality experienced by the British troops upon the western coast of Africa, and by the population of London when the plague rendered its habitations desolate!" 8.

Mr. Farr inquires,—To what is this excessive mortality referable, to the disease or to the treatment? In answer, he alludes to several facts.

1. The visiting justices of Hanwell state as "an extraordinary and disgraceful fact," that numbers of patients are sent into the asylum, as it would seem, to die. Of 656 deaths, 64 occurred within a month after admission. A similar complaint is made at many hospitals; and there is probably a tendency to send dangerous cases, or cases in their most critical stage, to public institutions. The exclusion of such cases from Bethlem reduces the mortality, but they cannot all be excluded without giving the asylums the advantages of that *selection,* which is so profitable to Assurance Offices. For in a disease so fatal as insanity, a certain number of lunatics are necessarily on the verge of death at the period of the disease when admission into an asylum is usually sought; and a due proportion of such cases cannot fairly be excluded.

Reference has also been made to the fact that out of 834 patients in Hanwell on December 31st, 1839, about 655 had been in other asylums, or workhouses, for considerable periods. Many cases were admitted in the chronic stages of insanity; but this, though it will account for a smaller number of recoveries, and the high proportion of fatal cases, will not account for a high *annual rate* of mortality. The *annual rate* of

* Medical Gazette, August 8th, 1835.

mortality is greater in the acute than in the chronic stage of insanity. Thus at the hospitals of Bethlem and St. Luke the annual mortality among the class called "curables" was 11 per cent., and only 6 per cent. among "incurables" (chronic cases). At Hanwell the annual mortality of lunatics in the state of mania, monomania, or melancholia appears, so far as it can be determined, to be about 12 per cent., while in cases of incoherence, imbecility, or dementia, (chronic stages of insanity,) about 8 per cent. die annually.

2. Mr. Farr pursues a batch of patients in the Hanwell Asylum through a period of 7½ years, and notes the deaths that successively occurred in each half year. The following are some of the main particulars.

The numbers stated to have been *relieved* were 14 per cent. of the numbers *cured and relieved;* and as the proportion remained nearly the same through the seven years, the two classes of facts have not been distinguished.

The annual rate of recovery in the *first half-year* was 24 per cent.; and the rate of mortality was nearly 25 per cent. The two rates remain high in the *second period* (the rate of recovery 16, and of mortality 14, per cent.), while they declined respectively to 3, and to 8 per cent. in the *third* period; and 2·3, and 7·0 per cent. annually, between the 5½ and 7½ years after admission into the asylum.

The rate of mortality, continues Mr. F., in an unit of time increases as the malady advances up to a certain point, and then declines regularly, in all diseases which have hitherto been investigated arithmetically. And this law regulates insanity. At Hanwell, 18 in 100 living die annually in the first 1½ year; and 8 in 100 annually for 6 years afterwards. If an asylum, therefore, contained none but persons in the first year and a half of the disease, (after admission is always understood,) the mortality would be 18 per cent.; while it would be 8 per cent. in an asylum for chronic cases between 1½ and 7½ years. Without implying any disparagement to the treatment in the former case, the rate of recovery in the two asylums would differ in a still greater degree, as it would be 19 per cent. in the first asylum, and only 3 per cent. in the second, set apart for the exclusive reception of the advanced cases. This separation seldom takes place in practice. The chronic and acute cases are always mixed in an institution like Hanwell; but it is evident that in the first years after it was opened, the proportion of cases in the early stages must have been greatest, and the proportion of lunatics in advanced periods of the disease must have since progressively increased. According to the above laws, the proportion of deaths and recoveries should gradually have declined, and this was the fact.

The annual mortality was 17 per cent. in the first three years, and 10 per cent. in the last three years; the annual rate of recovery was 14 per cent. in the first, and 8 per cent. in the last period. In the licensed houses which have been many years in existence, the annual rate of mortality was 13·6 per cent. in 1833-36, and 17·2 in 1836-39.

After some observations, Mr. Farr constructs a table of mortality and recovery for lunatics.

*Nosometrical Table.*

| No. | Period of the Disease dating from the day of Admission. | The Number of Lunatics who | | | Cases terminating in each Period. | | |
|---|---|---|---|---|---|---|---|
| | | Enter upon each Period. | Will Recover | Will Die Insane. | Total Number. | By Recovery. | By Death. |
| | Years. | *a* | *b* | *c* | *d* | *e* | *f* |
| 1 | 0 | 1,000 | 380 | 620 | 217 | 108 | 109 |
| 2 | 0·5 | 783 | 272 | 511 | 213 | 112 | 101 |
| 3 | 1·5 | 570 | 160 | 410 | 61 | 21 | 40 |
| 4 | 2·5 | 509 | 139 | 370 | 48 | 12 | 36 |
| 5 | 3·5 | 461 | 127 | 334 | 43 | 11 | 32 |
| 6 | 4·5 | 418 | 116 | 302 | 41 | 11 | 30 |
| 7 | 5·5 | 377 | 105 | 272 | 35 | 9 | 26 |
| 8 | 6·5 | 342 | 96 | 246 | 32 | 8 | 24 |
| 9 | 7·5 | 310 | 88 | 222 | .. | .. | .. |

If we take 1,000 lunatics at the stage of the disease corresponding to the time of admission at Hanwell, 217 will be discharged (108 recovered or relieved, and 109 dead) in the half-year following, leaving 783 to enter upon the second period, to be reduced year by year, until at the end of 7½ years only 310 remain. The range of the present series of observations extends no further, but the relative proportion of recoveries and deaths remains nearly as 88 to 222 during the last six years; and to complete the scheme of the table it may be assumed that 88 of the 310 will recover, and 222 will die. The columns *b*, *c*, shew, therefore, that of 1,000 cases, 380 will recover, and 620 die; that at the end of 1½ year, 160 will recover, and 410 will die.

The *mean future duration of insanity*, or *the expectation of disease*, cannot be deduced from the preceding table, because it breaks off at the end of 7½ years; but if the annual rate continued the same (1·10), 7 of 310 would remain insane 40 years, and the mean future duration of insanity at the period of admission at Hanwell would be 6·7 years; at the end of half a year it would be 8 years; and after 1½ year, it would be 10 years.

In the six years 1834-39, when the inmates were = 3,875 living 1 year, 706 were discharged; one in 5·5 therefore was discharged annually. If the institution had existed several years, and the numbers admitted and discharged had been equal, the mean duration would have agreed with this, and have been 5·5 years; but as Hanwell was opened in 1831, and only 1,179 out of 2,029 admitted, had been discharged on the 30th September, 1840, the 6·7 years is probably nearer the true mean duration.

Dr. Conolly ascertained the previous duration of the disorder in 191 cases (exclusive of 10 congenital cases) admitted during the year; 66 had been labouring under the disease less than six months; 26 between 6 and 12 months; 24 between 1 and 2 years; and 1 had been insane 39 years. The mean previous duration was 3·4 years. But, as little more than *half* the number had been insane *twelve months*, the time of admission may be represented by 1, or $1\frac{1}{4}$ year.

The mean age of 213 persons at admission was $36\frac{1}{2}$ years; the mean age of 195 at the time of the *first attack* of insanity was stated to be $32\frac{1}{2}$ years.

The *probable future duration of insanity* is shewn, by table, p. 27, to be $2\frac{1}{2}$ years at the time of admission; for, in $2\frac{1}{2}$ years, the 1,000 cases are reduced to 509. The chances that a patient will, or will not, remain insane $2\frac{1}{2}$ years are 509 to 491, or nearly equal. Among those who remain insane half a year after admission, the probable future duration of the disease is nearly 4 years.

The *probability of recovery* at admission $= \frac{380}{1000} = \cdot 380$; of dying insane $= \frac{620}{1000} = \cdot 620$. Half a year after admission the probability of recovery is $\frac{272}{783} = \cdot 347$; of dying insane $\frac{511}{783} = \cdot 653$. The numbers in juxta-position, in columns *b* and *c*, express the respective chances of death and recovery; thus, $5\frac{1}{2}$ years after admission at Hanwell, the chances are 272 to 105 that a lunatic will *not recover*. All these probabilities depend more or less on the assumption that 88 in 310, remaining at the end of $7\frac{1}{2}$ years, will ultimately recover.

The *probability of recovery, or of dying*, within any year, or years, up to $7\frac{1}{2}$ is accurately shewn by the table. In the first half-year the *probability of recovering* is $\frac{108}{1000} = \cdot 108$; the probability of recovering in $3\frac{1}{2}$ years is $\frac{380-127}{1000} = \frac{253}{1000} = \cdot 253$. Out of 1,000 cases, 253 recover in that time; hence $\cdot 253$ is the probability of recovery. The *probability of dying* in the first half-year is $= \frac{109}{1000} = \cdot 109$; in the two years following $\frac{511-370}{783} = \frac{141}{783} = \cdot 180$.

From a table of this kind the lives of lunatics can be insured; and, from the present table, they may be insured for a limited number of years.

The table is an instrument by which the effects of treatment on the mortality—the number of recoveries—and the duration of all diseases, can be accurately measured. It enables us to compare two or three different plans of treatment, and to determine their effects upon the principal results at which all medical treatment aims—the reduction of the mortality, and of the duration of the disease. Thus if 139, of 509 lunatics that have been $2\frac{1}{2}$ years in Hanwell, will recover under the present treatment, and 200 recover under any new mode of treatment that may be introduced, the advantages of the latter would be obvious; and still more so, if the *probable duration* of the disease were reduced from 10 to 5, or 2, years.

As *age* advances, the mortality and the probability of recovery decline. The sex, says Mr. Farr, age, and stage of the disease are the principal internal causes that influence the mortality, except the form of the disease which, exclusive of congenital idiocy, may be, perhaps, reduced to an element already discussed—the " stage of the disease." The influence of complications, of sex, and of age, may be assumed to be nearly the same in the licensed houses and Hanwell, as in ordinary asylums—the asylum, for instance, at Gloucester, where the mortality does not exceed 7 per cent. annually. The mortality of 7 per cent. may be fairly ascribed to insanity. The excess above this must be attributed to the diseases generated by the limited space in which the unhappy lunatics are confined—to the collection of large numbers under the same roof—the impurity of the atmosphere—the want of exercise and warmth—the poor unvaried diet—and the deficiency of medical attendance.* But the influence of these agents can only be ascertained by a Parliamentary inquiry; and it will not be denied that the causes should be investigated which raised the mortality of lunatics above the standard—57 per cent. among private patients, 71 per cent. at Hanwell, and 200 per cent. among paupers in the large licensed houses.

*Bethlem Hospital.*—This differs essentially from the Hanwell Asylum, as well as from the majority of the licensed houses, in the stricter selection of patients for admission. By the rules the following cases are inadmissible:—lunatics who have been insane for more than twelve months; who have been discharged uncured from other hospitals; in a state of idiocy; afflicted with palsy, or with epileptic, or convulsive, fits; and suffering from any dangerous disease. Notwithstanding the instructions in the admission papers, the petitions of 58 out of 311 (19 per cent.) who applied in 1836, were rejected. The patients are not allowed to remain longer than one year. 253 lunatics admitted in 1836 had been insane 83 days, on an average; 117 had been insane less than a month.

It would be exceedingly interesting to determine the mortality of this selected class of lunatics for 12 months. But, if dangerous symptoms come on at Bethlem, the patients are dismisssd, when practicable, as *improper objects*. Thus, of 3,026 discharged in 10 years, 829 were dismissed uncured, 483 as *improper objects*, and 145 dead. A great number of the "improper objects" would die soon after they left Bethlem; and their dangerous state, or supposed incurability, was the alleged cause of their dismissal. Paralysis, however slight, even of a finger, is the forerunner of death in the insane; and of 210 dismissed as improper objects (1831-36), 87 were paralytic, 59 "sick and weak," 24 epileptic, 4 apoplectic, 2 had "fits," and 28 were idiotic.

The lunatics at *Bethlem* are divided into three classes; "curables," "incurables," and "criminals."

21 of the lunatics on the list of the hospital were constantly out on leave of absence; and during the 13 years, 122 individuals were discharged as "out on leave of absence."

Of 100 "*curable*" patients discharged, 54·5 were cured, 5·2 died. The mean term of treatment was ·586 year, = 7 months; or ·49 year, = 6 months, if the time spent out of the hospital, on leave of absence, be excluded. The lunatics discharged as "improper objects" were 14·5 per cent.; a considerable portion of whom would have been numbered with the dead if they had remained.

The *annual* mortality was 8·8; the recoveries 92· per cent.; 24·5 per cent. were discharged as improper objects, 43·4 were discharged uncured;

---

* The diet and the condition of lunatics at Hanwell have been latterly ameliorated very considerably by the Visiting Justices, at the suggestion of the present accomplished physician; and the mortality may be expected to be reduced in proportion. It is also right to state that in some licensed houses the mortality of private patients does not exceed 7 per cent.

2·1 were out on leave of absence. 171 were discharged annually out of a constant population of 100.

If the deaths which occur among those out on leave of absence are not recorded, the annual mortality to 100 resident in Bethlem is 10·5.

*Incurables.*—72 "*incurables*" were admitted; 72 discharged (33 men, 39 women), and the average number resident for 13 years was 64·2. The years of life were therefore = 64·2 × 13 = 834. Nine incurables were cured, 39 died, and 24 were discharged at the request of their friends.

Of 100 cases 13 recovered, 33 did not recover, and 54 died. One in 11·6, = 6 per cent. were discharged annually; the mean term of residence was 11·6 years. 1 in 21, = 4·7 per cent. died, and 1 per cent. was cured annually.

"Incurables" is an improper term; but it is a recognition of the law that recovery is infrequent in advanced stages of insanity.

*Criminals.*—In the 13 years 71 criminal lunatics were admitted at Bethlem (56 men, 15 women); 51 were discharged, namely, 26 died, 2 escaped, and 23 recovered. The average number resident was 57·3, the years of life 745.

Of 100 cases, 45 recovered, 51 died. The annual rate of mortality was 3·5, of recovery 3·1 per cent.; the mean term of treatment deduced from the years of life, and the number discharged, was $14\frac{1}{2}$ years. The numbers admitted and discharged in the 11 years (1827-37) were nearly equal (36 and 39); and the years of life divided by the number discharged = $\frac{653}{39}$ = 16·7 years.

It is evident that several of the criminals, such as Oxford, cannot properly be said to labour under insanity—in the sense of a disease. It is, if anything, like idiocy, a congenital misdevelopment of the brain.

The number of recoveries is considerable at Bethlem, but less than at some private asylums, notwithstanding the careful selection of cases. The mortality is reduced by excluding dangerous cases, and by dismissing the patients on the verge of death, as "improper objects." It is difficult, under these circumstances, to account for the death of nine or ten in 100 annually, upon any other supposition than that the mortality is high at the early stage of the disease in Bethlem.

*Admission and Discharge of Lunatics.*—The last Committee of the House of Commons on Lunatics, stated in their report, "It has been clearly established in evidence, that there is no due precaution *with respect to the certificates of admission, to the consideration of discharge*, or to the application of any curative process, to the mental malady." Lunatics under confinement, it should be well recollected, are *prisoners;* and every one will admit that the depriving a man of his personal liberty, or turning loose a lunatic on society, are acts involving great responsibility,—a responsibility which, if it exist at all, is very imperfect in the present state of the law. In order to deprive a lunatic of his estates, a formal inquiry is publicly instituted; but a person who has been seven days chargeable to the parish may be committed as a lunatic to the County Asylum by two justices of the peace on the certificate of *any* physician, surgeon, or apothecary, asserting that the "said person *appears* to be insane of mind." 2,780 pauper lunatics are confined under these certificates in the county asylums.

But there are 1,389 lunatics, and 7,007 idiots, "under the care of the parish officers as in-door or out-door paupers." Many of them are necessarily under restraint, without either warrant or certificate; which is only required when the parishes think it necessary to send them to a public asylum, where their treatment costs two or three times as much as the workhouse fare.

Paupers may be sent to licensed mad-houses by a justice, or by the officiating clergyman and overseer, with one medical certificate; and other persons may be sent to a licensed house by any layman, upon the certificates of *any two* medical men. It appears also that by law, any person whom the governors choose to admit as a lunatic, may be confined at Bethlem, or St. Luke's Hospital, for an unlimited time.

The liberation of persons in confinement as lunatics, takes place under no better regulation. Medical visitors have been appointed, in the words of Lord Lyndhurst, "to see that the Chancery lunatics are well cared for, but above all *to watch the least glimmering of returning sanity, and see that the parties are not detained one day longer than necessary*." The relatives, parish-officers, proprietors, justices in petty-sessions, and the Metropolitan Commissioners release lunatics from the licensed houses; but the mode in which this is effected is by no means satisfactory. "When once," says Colonel Sykes, "they (pauper lunatics) get shut up in a mad-house, it is indeed difficult for them to regain their liberty." Lunatics are discharged at the discretion of the visiting justices from the county asylums; by the governors from Bethlem, St. Luke's, and other hospitals supported by subscription; and by the parish officers from workhouses.

Mr. Farr concludes—

"Many cases of abuse have occurred under the present system, which will be probably thought by the Society to require extensive alterations. And although there would be much difference of opinion on many points, all will probably agree that *no person should be placed under restraint as a lunatic in asylums, hospitals, or houses of any kind, who has not been examined by a public officer, practically acquainted with insanity*. I would therefore suggest that by some modification of the present system of inspection, the circumstances of every lunatic confined should be investigated personally by a crown officer, and recorded previous to committal, at the expiration of every quarter of a year after admission, and at the time of dismissal. The sex, and age, the stage, form and complications of insanity should be registered, on entering and leaving the several institutions, by impartial officers. This would be a protection to lunatics, and to the public; the deaths and recoveries would be registered on a uniform plan, and an invaluable statistical check on the results of treatment would be obtained."

